# Effects of Low‐Dose Cypermethrin Exposure on the Liver and Kidney of Swiss Albino Mice: Histopathological and Biochemical Insights

**DOI:** 10.1002/vms3.70706

**Published:** 2025-11-27

**Authors:** Swarup Kumar Kundu, Abu Sayeed, Solama Akter Shanta, Badhan Roy Tanny, Papia Khatun, Subarna Rani Kundu, Zahid Hasan Rocky, Md. Amim Al Maruf, Bishojit Bhattacharjee, Tonmoy Basu

**Affiliations:** ^1^ Department of Anatomy and Histology Faculty of Veterinary Animal and Biomedical Sciences Khulna Agricultural University Khulna Bangladesh; ^2^ Faculty of Veterinary Animal and Biomedical Sciences Khulna Agricultural University Khulna Bangladesh; ^3^ Department of Medicine Faculty of Veterinary Animal and Biomedical Sciences Khulna Agricultural University Khulna Bangladesh; ^4^ Department of Gynecology and Obstetrics Shaheed Tajuddin Ahmad Medical College Hospital Gazipur Bangladesh

**Keywords:** biochemical markers, histopathology, kidney, liver, low‐dose cypermethrin

## Abstract

Cypermethrin, a synthetic pyrethroid widely used in agricultural land areas and also for ectoparasitic control in livestock, has been implicated in various health effects. The study was conducted to elucidate the histopathological and biochemical consequences of low‐dose cypermethrin exposure in Swiss albino mice. A total of 30 mice were randomly divided into two groups: Control group (*n* = 15), fed standard diet and tap water, and treated group (*n* = 15), supplied with 0.12 mL/kg cypermethrin dissolved in 3 mL of drinking water once daily for 28 days. Histopathological evaluations of the liver and kidney, along with biochemical markers including aspartate aminotransferase (AST), alanine aminotransferase (ALT), blood urea nitrogen (BUN) and creatinine levels, were considered as indicators of low‐dose cypermethrin effects. The treated mice exhibited potential phenotypic characteristics including reduced body weight, shaggy fur and limb distention. Biochemical analysis revealed significant (****p < 0.0001) elevation of AST, ALT, BUN and creatinine values than control. Histopathological examination manifested severe damage to both organs. Hepatocyte hypertrophy, vacuolation, Kupffer cell proliferation, severe haemorrhage and disrupted sinusoidal architecture, infiltration of massive inflammatory cells, disappearance of normal hepatocytes found in liver. Renal tissues displayed glomerulosclerosis, tubular necrosis, loss of normal glomerular structure, proliferation of inflammatory cells and blackish discoloration of glomerulus. A significant (*p < 0.05) reduction of cell density (per unit of area) was also found in treated compared to control mice. In conclusion, this study suggests that exposure to cypermethrin at certain low dose causes histopathological and biochemical changes in the liver and kidney of Swiss albino mice.

## Graphical abstract

Impact of low‐dose cypermethrin exposure on health.

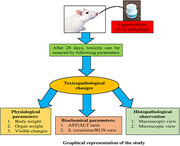

**Abbreviations**
ALT: Alanine aminotransferase
AST: Aspartate aminotransferase
BUN: Blood urea nitrogen
GABA: Gamma‐aminobutyric acid
ICDDR'B: International Center for Diarrheal Disease Research,Bangladesh

## 1 Introduction

 Pesticides are structurally chemical substances that are rapidly used to control unwanted products such as fungi, insects, etc. on agricultural land (Seven et al. 2022). Various organisms mainly invade land and disrupt productivity. So, the sustainable production of agricultural land largely depends on the use of various pesticides (Shammi et al. 2020). According to a study of Bangladesh Rice Research Institute (BRRI), farmers have been increasing the use of different pesticides day by day and its long‐term residual effects are causing serious health risks like dermatologic problems, genetic disorders, renal dysfunction, high blood pressure, infertility, bradycardia (Ali et al. 2020). Occupational exposure of farm workers to pesticides through skin or close contact, inhalation, ingestion or other routes may cause the aforementioned health hazards (Seven et al. 2022). In addition, the human population is exposed to some chemical pesticides in their daily routine through food, beverages, cosmetic products and indoor and outdoor pollutants (Priyanka et al. 2020). Cypermethrin is a type‐II synthetic pyrethroid with higher potency than other broad‐spectrum insecticides also used to control ectoparasitic infections in the livestock sector (Shuklan et al. 2023). The lipophilic nature of cypermethrin allows it to penetrate cell membranes and damage internal structures. Also being hydrophobic in nature, different concentrations were identified in different organs (Manna et al. 2004). The highest concentrations were recorded in the fat, liver, kidney, brain tissue, and blood samples (G El‐Shemi and Abou El‐Ella 2015). Acute exposure to cypermethrin significantly alters haematological parameters leading to major health complications. Although the absorption of cypermethrin into the blood is low, but it is rapidly metabolized in the liver and excreted in the urine (Manna et al. 2005). Elevation of BUN or serum creatinine level not only indicates kidney dysfunction, but their ratio also accurately amplifies the structural and functional abnormalities of the kidney (Baum et al. 1975). Similar to these, liver enzyme markers ALT, and AST are found in the blood in large amounts during hepatic function disturbances, and high levels of these markers indicate hepatic injury (Abdul‐Hamid et al. 2017). Among the severe harmful effects of cypermethrin on vital organs, histological changes such as hyperplasia, necrosis, destruction of alveolar cells in lung tissue, tubular inflammation, glomerular deformation, cellular infiltration around central and portal veins, destruction of hepatocytes, haemorrhage in the myocardium, spermatogenesis inhibition, nerve and brain damage are observed in the studies of different researchers (Grewal et al. 2010; Sheikh et al. 2014). Furthermore, cypermethrin is approved for use in the livestock sector. It inhibits reproductive function and disrupts the transmission of nerve impulses by blocking sodium ion channels in nerve cell membranes (Nasuti et al. 2007). Even ingestion of cypermethrin can cross the blood–brain barrier and induce neurotoxicity and motor dysfunction through modulation of gamma‐aminobutyric acid (GABA) levels or increase in free radicals (Seven et al. 2022). According to the above previous statement by different researchers on cypermethrin toxicity, the present author designed the proposed research work based on the following potential objectives:

- To assess the potential visible abnormalities in health of low‐dose cypermethrin‐exposed mice
- To determine hepatic and renal damage by evaluating histopathological changes and biochemical markers in low‐dose cypermethrin‐exposed mice

**Highlights**

- Low‐dose cypermethrin exposure in mice results in several abnormal phenotypic traits, including hind limb dragging, itchy skin, abortion, reduced growth,s etc.
- Potential histopathological alterations are observed in hepatic and renal tissues.
- Biochemical assessments reveal changes in markers such as AST/ALT ratio and the BUN to serum creatinine ratio, further confirming the presence of hepatic and renal dysfunction.
- To mitigate potential health risks, it is recommended to avoid acute exposure to cypermethrin in daily life.

## 2 Material and Methods

### 2.1 Test Material and Animal Care

A total of 30 healthy experimental Swiss albino mice of both sexes aged 7–8 weeks were purchased from International Center for Diarrheal Disease Research (ICDDR'B), Mohakhali, Dhaka, Bangladesh. Collected mice were closely observed for finding abnormalities with the naked eye to ensure that each mouse was suitable for the purposes of the present study. The initial weight of each mouse was taken using a standard analytical balance (Balance LX200ABL) and recorded as approximately 32 ± 5 g. Before the commencement of the experiment, mice were kept in the Anatomy laboratory under the mentioned condition. Regular cleanliness and proper hygiene were maintained in the mice‐rearing box as well as the laboratory premises to keep the animals in both disease and stress‐free conditions. 12/12‐h light/dark cycles, the 20°C –22°C room temperature was carefully maintained and normal mice pellet was provided with fresh drinking water three times a day for 7 days.

### 2.2 Collection of Cypermethrin

Cypermethrin was sourced from the local market within the study area. Various formulations of cypermethrin were used by local farmers, with veterinary‐grade cypermethrin being the primary product used in our study. This veterinary‐grade cypermethrin is commonly employed for ectoparasitic control in livestock. For our research, we specifically used a 12% concentration of cypermethrin (Pharmacodynamic Influential Factors: Animal species, form: Liquid, colour: Light yellow, trademark: BGKLH), following the application protocol recommended by the manufacturer.

### 2.3 Cypermethrin Dose Selection and Calculation

In previous studies of different experts or chemists, various doses of cypermethrin were used (Seven et al. 2022) but 0.12 mL/kg/day was specifically chosen for this study to reflect low exposure scenario that is relevant to both environmental and occupational conditions involving cypermethrin. The dose chosen is within a range that allows investigation of potential toxic effects and is comparable to the level of exposure that may be encountered by humans or livestock under certain circumstances. Cypermethrin is usually used in livestock, in the form of sprays for flea/tick treatments. Although human exposure is typically low, it can expose through dermal contact, inhalation or ingestion. A dose of 0.12 mL/kg/day is a reasonable approximation of the type of exposure that may be seen in cases of accidental or repeated, low‐level exposures handling pesticides or living in agricultural areas where pyrethroid use is common. Besides this, livestock, particularly cattle, poultry and pets, are gradually exposed to pyrethroids through veterinary treatments. A dose of 0.12 mL/kg/day in mice reflects an exposure level that animals may encounter in the environment, where pyrethroid‐based treatments are applied. According to the selection dose of 0.12 mL/kg/day, every mouse was (standard weight for every mouse was recorded at 32 g) exposed to 0.00384 mL cypermethrin orally once daily for 28 days.

### 2.4 Study Protocol and Tissue Processing

After proper acclimatization to the above‐mentioned conditions in anatomy laboratory, collected mice (*n* = 30) were randomly divided into two groups (control and treated), 15 mice were in each group.

**Control**: Standard pellet feed + fresh drinking water
**Treated**: Standard pellet feed + cypermethrin (at a dose of 0.12 mL/kg) was mixed with 3 mL of drinking water for each mouse and fed orally by flexible gavage needle once daily for 28 days.

In the meantime, of study tenure, any visible abnormalities like hyperexcitability, regular posture, any wounds or skin infections or allergic conditions, urination, mice droplets, regular feed intake, and movement activity were closely monitored. Therefore, at the end of the experimental period (28 days), all mice were sacrificed ethically. First every mouse was deeply anaesthetized by injecting sodium pentobarbital (80 mg/kg) intraperitoneally and then perfused with PBS solution and 4% PFA. Liver and kidney samples were then collected and fixed overnight in 4% PFA solution. The sample was again fixed overnight in 20% sucrose solution. Finally, paraffin embedding was performed and the tissue was sectioned at 5 µm thickness using a rotary microtome (Epredia^TM^ HM 325, THERMO FISCHER Scientific). Tissue processing and staining procedures were maintained according to the guidelines of (Kundu et al. 2023; Cardiff et al. 2014). The required images were taken at 200 and 50 µm focus using a photomicroscope (Model: SKU: B120C‐E520200610, AMSCOPE LOS ANGLES USA). Finally, total viable cells were counted for hepatic and renal tissue sections using Image J 1.54 g (Wayne Rasband and contributor National Institutes of Health, USA). Cells per unit of area or cell density was calculated by the following formula:

Cells per unit of area or cell density = Total number of cells ​/Total area (square unit)

### 2.5 Physiological Parameters Analysis

After starting the experiment, the body weight of each mouse was measured using a standard analytical balance (LX200ABL) on the initial day (Day‐1), Day‐7, Day‐14, Day‐21 and Day‐28. Feed intake, normal movement, external visual changes or any other detectable abnormalities were recorded as toxicity parameters of low‐dose cypermethrin exposure.

### 2.6 Serum Preparation and Biochemical Markers Analysis

For biochemical markers analysis, about 1.5 mL of blood sample was collected directly from the left ventricle and kept in a blood collection tube without adding anticoagulant for serum preparation. The blood containing tube was placed in upright slanting position at room temperature for 6 h. They were then incubated overnight in the refrigerator (4°C). The serum samples were separated by centrifugation and collected by using 200 µL pipette. Serum sample were stored in capped tube at −20°C for biochemical study. Biochemical analysis (ALT, AST, S. Creatinine and BUN) was done by using Stat Fax‐3300 Semi Auto Chemistry Analyzer. L‐Alanine, L‐Aspartate and alpha‐ketoglutarate were used for ALT and AST assay kit. After obtaining the values of the following markers, the data was carefully examined and following formula was used to determine the AST/ALT and BUN/S. Creatinine ratio.

$$\begin{array}{c} \begin{array}{c} \mathrm{AST}/\mathrm{ALT}\;\mathrm{ratio}=\mathrm{ALT}\;\mathrm{level}\;\left(U/L\right)÷\mathrm{AST}\;\mathrm{level}\;\left(U/L\right)\\ \mathrm{BUN}/S.\mathrm{Creatinine}\;\mathrm{ratio}=\mathrm{BUN}\;\mathrm{level}\;\left(\mathrm{mg}/\mathrm{dL}\right)\;\\ ÷\;\mathrm{Serum}\;\mathrm{Creatinine}\;\mathrm{level}\;\left(\mathrm{mg}/\mathrm{dL}\right) \end{array} \end{array}$$

### 2.7 Ethical Permit

The experiment was conducted following the animal welfare and experimentation ethics guidelines of Bangladesh Agricultural University. Before the experimental work, the study protocol was approved by the Animal Welfare and Experimentation Ethics Committee (approval number AWEEC/BAU/2023(35)).

### 2.8 Statistical Evaluation

Statistical analysis of the data obtained from the present experiment was done by using GraphPad Prism 9 (GraphPad Software, Inc., San Diego, CA, USA) software. Significance assessment was conducted using an unpaired *t*‐test. Results were shown as mean ± SEM. (*) and (****) shows a statistically significant difference at the *p* < 0.05 and *p* < 0.0001 level. Moreover, ns denotes the statistically non‐significant difference between control and treated values.

## 3 Results

### 3.1 Effects of Low‐Dose Cypermethrin on Health

#### 3.1.1 Body Weight

**FIGURE 1:**
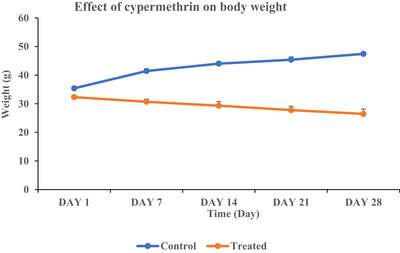
The body weight of mice in the cypermethrin‐exposed group decreased significantly (****p < 00.0001) over 28 days. Weight loss followed a consistent pattern in the exposed group each week, whereas the control group did exhibit a consistent trend of weight gain. Data are expressed as mean ± SEM.

According to the data from the initial day to the 28th day, the body weight of the cypermethrin‐treated group of mice was significantly (****p < 0.0001) decreased (from 34.23 ± 0.253 g to 29.45 ± 0.31 g). In the control group, initial (Day‐1) body weight was recorded as 35.75 ± 0.257 g which increased significantly (****p < 0.0001) on day of 28 at 47.44 ± 0.19 g. Compared to the control group, the treated group of mice showed a significant decrease of 37.92% body weight at the end of experiment (Table 1 and Figure 1).

**TABLE 1:** Cypermethrin exposure gradually reduce the body weight condition day by day.

Group	Day‐1 (Initial)	Day‐7	Day‐14	Day‐21	Day‐28
Control	35.75 ± 0.257 g	41.45 ± 0.351 g	44.02 ± 0.338 g	45.39 ± 0.44 g	47.44 ± 0.19 g
Treated	34.23 ± 0.253 g	33.19 ± 3.47 g	32.24 ± 1.50 g	32.15 ± 1.34 g	29.45 ± 0.31 g

#### 3.1.2 Organs Weight

**FIGURE 2:**
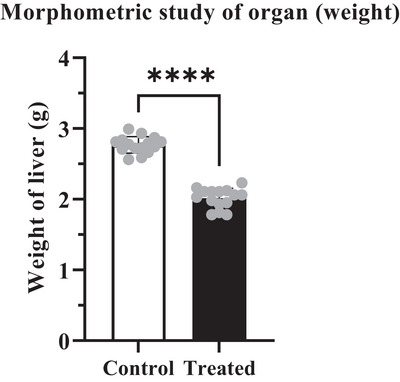
Quantitative analysis revealed a significant reduction in liver weight in the cypermethrin‐exposed treated group. The dots in the columns represent the standard deviation for both the control and treated groups. The (****) symbol indicates a statistically significant difference at the *p* < 0.0001 level.

**FIGURE 3:**
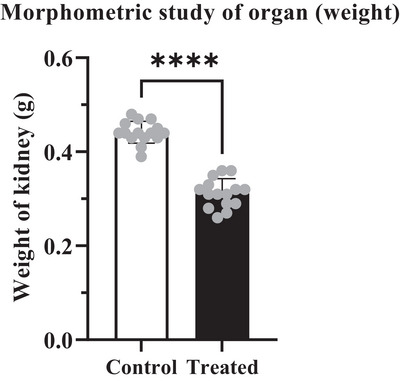
Quantitative analysis revealed a significant reduction in kidney weight in the cypermethrin‐exposed treated group. The dots in the columns represent the standard deviation for both the control and treated groups. The (****) symbol indicates a statistically significant difference at the *p *< 0.0001 level.

Analysis of the weight of different organs (liver and kidney) showed significant variation (****p < 0.0001) between control and treated group of mice. According to Tables 2 and 3, the weight of the liver and kidney in the control group of mice represented 2.81 ± 0.024 g and 0.438 ± 0.009 g. Whereas in the treated groups, it presented a reduced value of 2.00 ± 0.053 g and 0.302 ± 0.019 g respectively which carried a statistically significant difference (****p < 0.0001) (Figures 2 and 3). The data also represented a weight loss of 28.83% in liver and 31.05% in the kidney at the treated group (Figures 2 and 3).

**TABLE 2:** Liver weight of control and cypermethrin‐treated mice after 28 days.

Groups	Control	Treated	*p*‐value
Weight of liver (mean ± S.E.M.)	2.769 ± 0.024 g	2.015 ± 0.053 g^****^	<0.0001

**TABLE 3:** Kidney weight of control and cypermethrin‐treated mice after 28 days.

Groups	Control	Treated	*p*‐value
Weight of kidney (mean ± S.E.M.)	0.4413 ± 0.009 g	0.3120 ± 0.019 g****	<0.0001

### 3.2 Effects of Low‐Dose Cypermethrin on Physiological Parameters

During the study period, some considerable physical abnormalities were recorded. Mice in the cypermethrin‐exposed group had distended limbs, reluctance to move, abnormal defecation and urination, red spots on the skin, lethargy, rapid abortion. Normal body growth, regular feed consumption and alertness were seen in the control group of mice. Immediate after every meal, they were itching by themselves in their throat region by extending their limb (Table 4).

**TABLE 4:** Experimental condition of mice in the observation period.

Parameters	Control	Treated
Movement	Normal posture	Tendency towards immobility
Feed intake	Regular	Reduced^*^
Limb distention	−	+
Alertness	+	−
Activeness	+	‐ (lethargy)
Dermatologic condition	Normal, no itching	Red spot with itching
Body growth	↑	↓^*^
Droplets	Regular (approx. 57/day)	Abnormal (approx. 21/day)
Defecation	Regular and normal	Irregular and sometimes fluid
Hair types	Short, sleek and shiny	Messy hair
Abortion	−	+

### 3.3 Effects of Low‐Dose Cypermethrin on Biochemical Markers

#### 3.3.1 AST/ALT and S. Creatinine/BUN Ration

**FIGURE 4:**
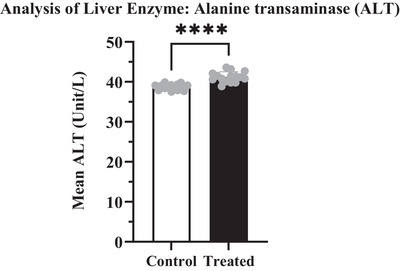
ALT level in the treated group (41.19 U/L) was significantly (****p < 0.0001) higher than those in the control group (38.73 U/L), with a difference of about 2.460 ± 0.3901. At 95% confidence interval the upper and lower limit variation for control and treated groups were found 1.661 to 3.259.

**FIGURE 5:**
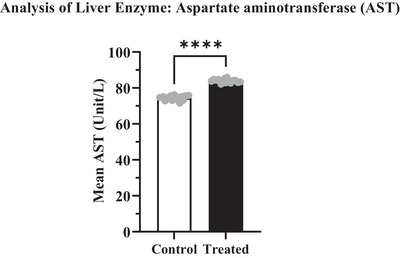
AST level in the treated group (83.97 U/L) was significantly (****p < 0.0001) higher than those in the control group (74.43 U/L), with a difference of about 9.540 ± 0.4244. At 95% confidence interval the upper and lower limit variation for control and treated groups were found 8.671 to 10.41.

**FIGURE 6:**
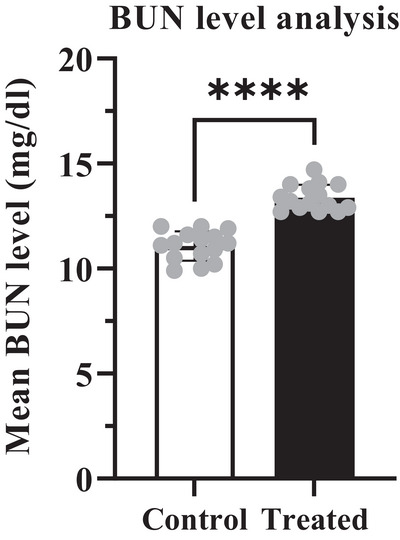
Mean value of BUN in the treated group (13.38 mg/dL) was significantly (****p < 0.0001) increased than the control group (11.07 mg/dL), with a difference of about 2.307 ± 0.2402. At 95% confidence interval the upper and lower limit variation for control and treated groups were found 1.815 to 2.799.

**FIGURE 7:**
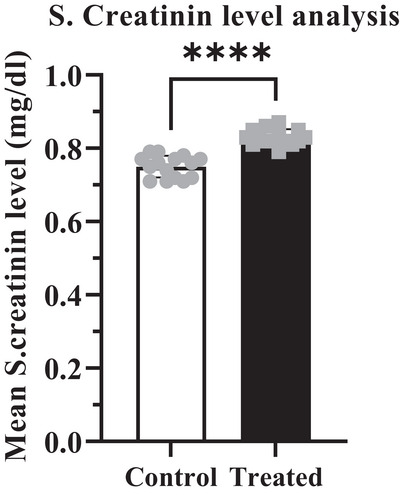
Mean value of Serum creatinine in the treated group (0.8307 mg/dL) was significantly (****p < 0.0001) increased than the control group (0.7500 mg/dL), with a difference of about 0.08067 ± 0.009384. At 95% confidence interval the upper and lower limit variation for control and treated groups were found 0.06144 to 0.09989.

The potential effects of cypermethrin on some targeted biochemical parameters have been shown in Table 5. Depending on the administration of cypermethrin in the treated group, enzymatic markers like ALT, AST, BUN and S. creatinine levels were increased compared to the control group (Figures 4, 5, 6, 7). The highest values were recorded in the treated group for both enzymatic parameters including ALT (40.62 ± 0.307 U/L) which was an increase of about 3.15% (Figure 4) and AST (84.04 ± 0.216 U/L) which was an increase of about 11.25% (Figure 5) compared to the control value. According to data from BUN and S. creatinine values, BUN level (13.04 ± 0.133 mg/dL) increased by approximately 16.43% (Figure 6) and S. creatinine level (0.81 ± 0.007 mg/dL) also increased by approximately 6.58% (Figure 7) in the treated group than the control. Moreover, the AST/ALT ratio was recorded as >2, and the ratio from BUN to creatinine was found >15 which significantly (****p < 0.0001) higher than the control level.

**TABLE 5:** Changes of different biochemical parameters in the experimental mice exposed to cypermethrin.

Parameters	Control	Treated	*p*‐value
ALT (U/L)	38.73 ± 0.199	41.19 ± 0.307****	<0.0001
AST (U/L)	74.43 ± 0.242	83.97 ± 0.216****	<0.0001
BUN (mg/dL)	11.07 ± 0.192	13.38 ± 0.133****	<0.0001
S. creatinine (mg/dL)	0.7500 ± 0.014	0.8307 ± 0.007****	<0.0001

### 3.4 Histopathological Observation in Low‐Dose Cypermethrin Exposure

**FIGURE 8:**
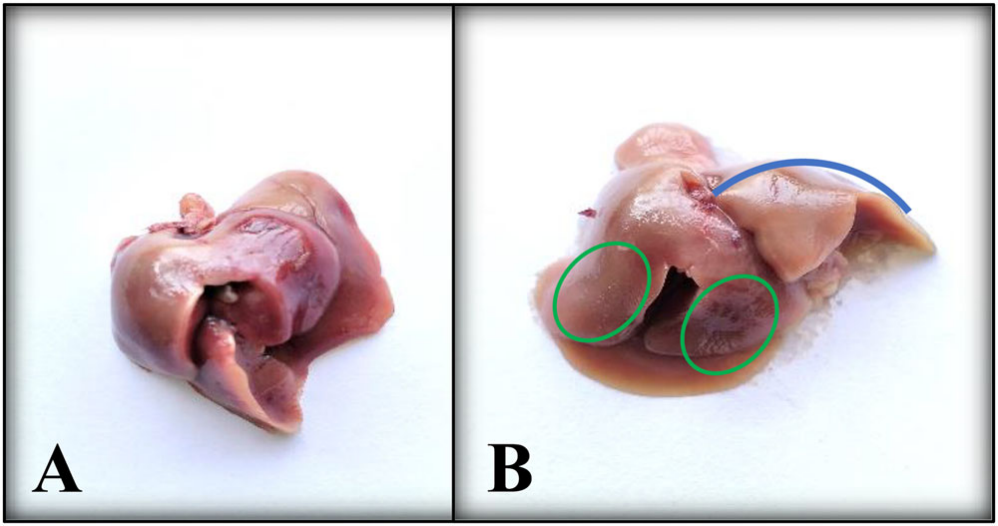
**(A, B)**: Macroscopic view of liver. A: Control, B: Treated. Representative photographs of the liver show well‐defined, red, glossy lobes in the control group, while the treated group exhibits fragile, fatigued lobes with discoloration (blue curve) and mild hemorrhaging (green oval).

**FIGURE 9:**
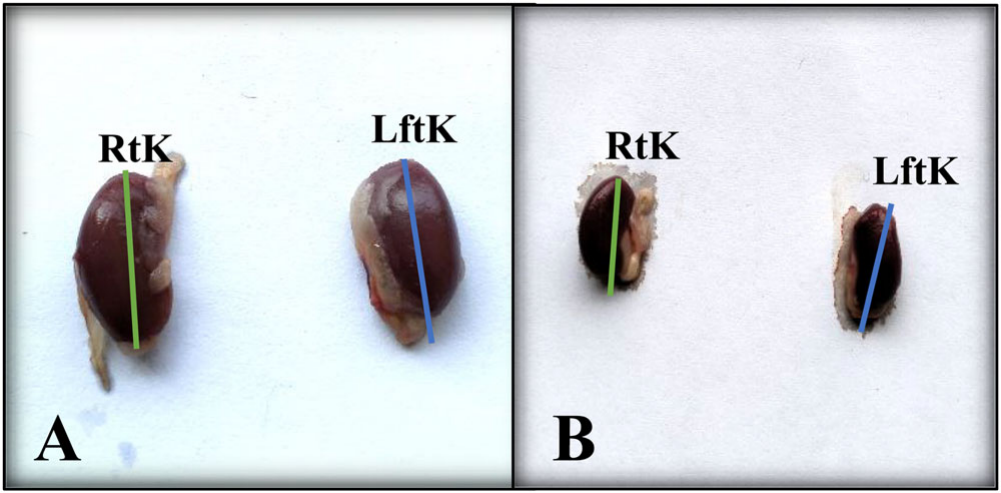
**(A, B)**: In the macroscopic examination of the kidneys, the control group (A) exhibited normal renal architecture with no noticeable changes in size or appearance. In contrast, the treated group (B) demonstrated a slight reduction in kidney size, accompanied by a bright red appearance, indicating potential alterations in the tissue. RtK‐Right Kidney, LftK‐Left Kidney.

**FIGURE 10:**
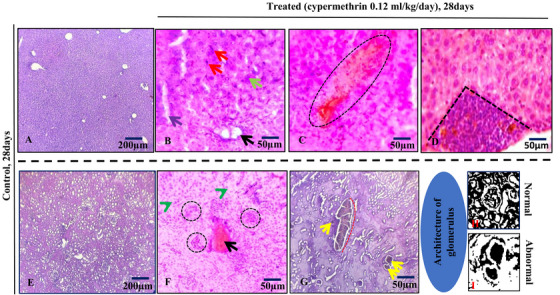
Transverse section of representative photomicrographs of liver tissue(A–D) show several pathological alterations in the treated group of mice. These include large vacuoles (black arrow), proliferation of Kupffer cells (red arrow), severe hemorrhagic areas (black rounded dot), deformities in the sinusoidal space (blue arrow), hypertrophied hepatocytes (green arrow), and aggregation of inflammatory cells (black dashed line). Additionally, there is a disappearance of normal hepatocytes in the treated group. Photomicrographs of renal tissue (E–G) also illustrate the various structural changes in the treated group. These include clusters of inflammatory cells (rounded dot), severe hypertensive glomerulosclerosis (red arrow), severe tubular necrosis (arrowhead), and loss of proper glomerular architecture. The glomerulus exhibits blackish discoloration (yellow arrow) along with extensive hemorrhage and enlargement of the tubular portion (red dashed line). Notably, (H, I) the glomerular structure severely affected (fine‐grained images) in the treated mice than control. Staining: H&E. Magnification bar: 200 µm and 50 µm.

**FIGURE 11:**
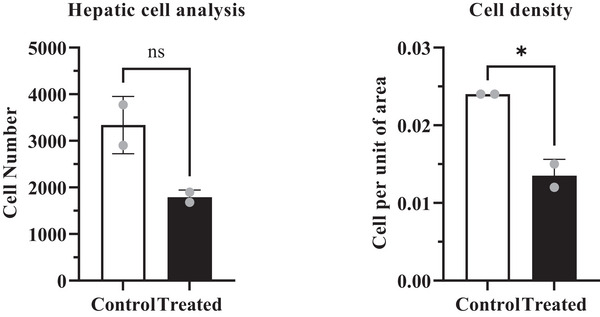
Quantitative analysis of total hepatic Cell Count and cell density in Control and cypermethrin‐treated Mice. The (*) symbol indicates a statistically significant difference at the *p* < 0.05 level. ns, non‐significant.

**FIGURE 12:**
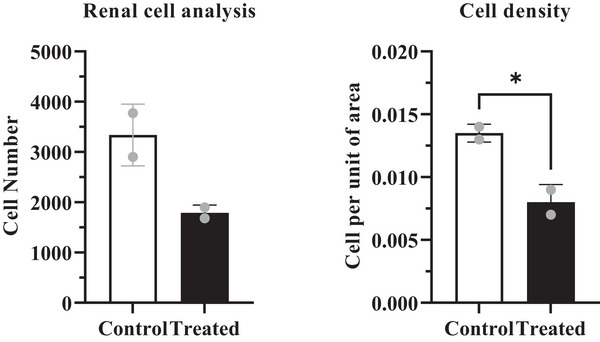
Quantitative analysis of total hepatic Cell Count and Cell Density in Control and Cypermethrin‐Treated Mice. The (*) symbol indicates a statistically significant difference at the *p* < 0.05 level. ns, non‐significant.

Exposure to cypermethrin causes hepatotoxicity and reno toxicity which were confirmed by yellowish, mild discoloured, extreme fragile lobes, haemorrhagic and fatigued liver (Figure 8), as well as slight reduction of size and dark colouration of the kidney surface (Figure 9) in the macroscopic view. Transverse section of liver (Figure 10) showed large vacuoles in some hepatocytes, Kupffer cells proliferation, severe haemorrhagic area in portal vein, deformities in sinusoidal space, hypertrophied hepatocytes and aggregation of inflammatory cells in the cypermethrin exposed group of mice. In kidney tissues of cypermethrin exposed mice (Figure 10), photomicrographs also showed cluster of inflammatory cells in the entire tissue, severe hypertensive glomerulosclerosis and severe tubular necrosis, blackish discoloration in the glomerulus, extensive haemorrhage and enlargement of the tubular portion. Abnormal glomerular structure detected in fine‐grained images of kidney pathology treated with cypermethrin. The number of viable cells was reduced in both hepatic and renal tissues of the group of mice exposed to cypermethrin (Figures 11 and 12; Table S1). A significant (*p < 0.05) reduction of cell number in per unit of area was shown in treated compared to control mice (Figures 11 and 12).

## 4 Discussion

The present study demonstrates significant health hazards in low‐dose cypermethrin exposure, particularly in relation to body weight loss, organ damage, elevation of biochemical markers and histopathological alterations in Swiss albino mice. Cypermethrin at low‐dose (12 mL/kg) exposure led to a notable loss in body weight (37.92%). Oral exposure of cypermethrin at a dose of 25 mg/kg exhibits preliminary toxic effects on weight (Adjrah et al. 2013). In the current study, liver and kidney weights were significantly reduced by 28.83% and 31.05%, respectively, in the cypermethrin‐treated group compared to controls. This is in contrast to the findings of Sangha et al. (2011), who reported a significant increase in kidney weight (21.22%) following cypermethrin exposure. At the administered dose (12 mL/kg) of cypermethrin, few considerable physiological changes in the mice, including lethargy, dragging of the right hind limb, reluctance to move and irregular urination, defecation were noticed. In addition, some animals exhibited red spots on their skin, and there were reports of early miscarriages. These symptoms align with mild toxic symptoms at doses of 35 and 50 mg/kg of cypermethrin, such as diarrhoea, ataxia and salivation (Grewal et al. 2010; Shilpakar and Karki 2021). Biochemical markers of liver and kidney were significantly elevated in the low‐dose cypermethrin‐exposed mice, as evidenced by increased levels of ALT, AST, BUN and serum creatinine. These markers are commonly associated with liver and kidney dysfunction (Amir and Amir 2015; Wibowo et al. 2008). AST, normally localized in the liver and released into the bloodstream during hepatic injury, makes it a reliable marker of liver damage. Similarly, ALT elevation reflects hepatocellular damage and supports the toxic effects of cypermethrin on the liver. As for renal function, elevated levels of BUN and serum creatinine are indicators of kidney dysfunction, as both are primarily excreted through the kidneys (Seven et al. 2022). AST/ALT ratio greater than 2 is indicative risk factor of hepatocellular injury, which was also observed in the current study (Chen et al. 2022). Moreover, if the AST/ALT ratio is less than 1, that indicates the level of mild hepatic injury, and if the ratio exceeds the value 1, it considers severe hepatic damage (Scheipner et al. 2021). In contrast, an excessive BUN to S. creatinine ratio is an indication of either impaired renal tissue function or dehydration of the body (Huang et al. 2022). Histopathological examination of liver at a relatively low dose of 0.12 mL/kg, exhibited signs of cellular degeneration, including hepatocyte vacuolization, congestion in the central and portal veins and Kupffer cell proliferation, that are similar to the changes at different higher doses (Yavasoglu et al. 2006). Thickening of the pyknotic nucleus and malformed hepatic plates also found in animals exposed to 50 and 75 mg/kg of cypermethrin (Shuklan et al. 2023). The liver is a key organ involved in detoxification, and it plays a central role in metabolizing toxic substances, including pesticides like cypermethrin. When cypermethrin is absorbed into the bloodstream, it is metabolized in the liver by enzymes, leading to the formation of potentially reactive intermediates. These intermediates can bind to cellular macromolecules, causing oxidative stress, lipid peroxidation and cellular damage. In the kidneys, histopathological changes included hypertensive glomerulosclerosis, accumulation of inflammatory cells, severe tubular necrosis and deformities in the glomerular architecture have also been appeared. Glomerulonephritis, renal tubular dilatation and cellular infiltration are the common effects in high dose of cypermethrin‐exposed mice (Shuklan et al. 2023; Grewal et al. 2010; Shivanoor and David 2014). The accumulation of cypermethrin in renal tissue likely contributes to all of these toxic effects. Metal‐binding protein metallothionein, which accumulates in response to cypermethrin exposure, may impair organelle function in the kidneys, further exacerbating tissue damage (Prashanth 2011). In addition, cypermethrin has been shown to increase biomarkers (BUN and creatinine) of serum biochemical levels which indicative to kidney injury. These elevated levels suggest impaired renal filtration and indicate that cypermethrin exposure can lead to both acute and chronic kidney dysfunction. Furthermore, the current study revealed that exposure to cypermethrin caused a reduction in kidney size, accompanied by noticeable liver fragility, findings that mirror the observations of Vardavas et al. (2016), who reported similar pathological changes following cypermethrin exposure. These results further support the notion that cypermethrin exerts detrimental effects on both hepatic and renal tissues, even at relatively low doses. In summary, our study highlights the significant health risks associated with low‐dose cypermethrin exposure, demonstrating both biochemical and histopathological evidence of liver and kidney damage. The observed effects, including weight loss, altered biochemical markers and tissue damage, underscore the potential toxicity of cypermethrin, particularly at environmentally relevant doses.

## 5 Conclusion

The widespread use of cypermethrin has raised significant concerns regarding its potential health risks. Toxic effects caused by exposure to low doses of cypermethrin disrupt key serum biochemical parameters, including AST, ALT, creatinine and BUN, while simultaneously leading to pathological changes in liver and kidney tissues. Further research is needed to better understand the mechanisms of toxicity by using antibody markers and to establish safe exposure limits.

## Author Contributions

**Swarup Kumar Kundu**: conceptualization, data curation, formal analysis, funding acquisition, investigation, methodology, project administration, resources, software, supervision, validation, visualization, writing – original draft, writing – review and editing. **Abu Sayeed**: data curation, formal analysis, investigation, software, visualization. **Solama Akter Shanta**: investigation, methodology, supervision, validation. **Badhan Roy Tanny**: formal analysis, writing – original draft, writing – review and editing. **Papia Khatun**: formal analysis, writing – review and editing. **Subarna Rani Kundu**: formal analysis, writing – review and editing. **Zahid Hasan Rocky**: formal analysis, software. **Md. Amim Al Maruf**: formal analysis, software. **Bishojit Bhattacharjee**: formal analysis, software. **Tonmoy Basu**: formal analysis, software.

## Funding

Special thanks to the Khulna Agricultural University Research System (KAURES) (FY:2022‐2023) (SL No. 06) for proving the financial support to conduct the research efficiently.

## Conflicts of Interest

The authors declare no conflicts of interest.

## Supporting information




**Supplementary File 1**: Supplementary Table. 1

## Data Availability

The datasets generated the current study are available from the corresponding author upon reasonable request.
