# Supplementary material for: Effects of Low‐Dose Cypermethrin Exposure on the Liver and Kidney of Swiss Albino Mice: Histopathological and Biochemical Insights
Source: Vet Med Sci. 2025 Nov 27;12(1):e70706. doi: 10.1002/vms3.70706 (PMC12658335; doi:10.1002/vms3.70706)
Supplement: Supplementary file 1 — Supplementary Table. 1 [file VMS3-12-e70706-s001.docx]

**Supplementary**

**Table. 1**

| **Group** | **Slide no.** | **Hepatic Cell Count** | **Total area** | **%Area** | **Mean** | **Cell per unit of Area** | **p-value** |
| --- | --- | --- | --- | --- | --- | --- | --- |
| **Control** | L-1001 | 4475 | 185879.98 | 6.879 | 255 | 0.024 | 0.0742 |
|  | L-1002 | 4759 | 197676.61 | 7.315 | 271 | 0.024 |  |
| **Treated** | L-2001 | 2575 | 175861.34 | 3.958 | 146 | 0.015 | 0.0198* |
|  | L-2002 | 2234 | 186112.54 | 3.434 | 127 | 0.012 |  |

| **Group** | **Slide no.** | **Renal cell Count** | **Total area** | **%Area** | **Mean** | **Cell per unit of Area** | **p-value** |
| --- | --- | --- | --- | --- | --- | --- | --- |
| **Control** | K-3001 | 2904 | 202392.054 | 6.701 | 255 | 0.014 | 0.0742 |
|  | K-3002 | 3772 | 286847.065 | 9.585 | 255 | 0.013 |  |
| **Treated** | K-3003 | 1897 | 206312.965 | 6.812 | 255 | 0.009 | 0.0389* |
|  | K-3003 | 1680 | 256786.324 | 6.345 | 255 | 0.007 |  |
